# Present Impact of AlphaFold2 Revolution on Structural Biology, and an Illustration With the Structure Prediction of the Bacteriophage J-1 Host Adhesion Device

**DOI:** 10.3389/fmolb.2022.907452

**Published:** 2022-05-09

**Authors:** Adeline Goulet, Christian Cambillau

**Affiliations:** ^1^ Laboratoire D’Ingénierie des Systèmes Macromoléculaires (LISM), Institut de Microbiologie, Aix-Marseille Université—CNRS, Marseille, France; ^2^ School of Microbiology, University College Cork, Cork, Ireland

**Keywords:** AlphaFold2, structural biology, bacteriophage, host adhesion device, bacteriophage-host interactions, *Lactobacillus casei* bacteriophage J-1

## Abstract

In 2021, the release of AlphaFold2 - the DeepMind’s machine-learning protein structure prediction program - revolutionized structural biology. Results of the CASP14 contest were an immense surprise as AlphaFold2 successfully predicted 3D structures of nearly all submitted protein sequences. The AlphaFold2 craze has rapidly spread the life science community since structural biologists as well as untrained biologists have now the possibility to obtain high-confidence protein structures. This revolution is opening new avenues to address challenging biological questions. Moreover, AlphaFold2 is imposing itself as an essential step of any structural biology project, and requires us to revisit our structural biology workflows. On one hand, AlphaFold2 synergizes with experimental methods including X-ray crystallography and cryo-electron microscopy. On the other hand, it is, to date, the only method enabling structural analyses of large and flexible assemblies resistant to experimental approaches. We illustrate this valuable application of AlphaFold2 with the structure prediction of the whole host adhesion device from the *Lactobacillus casei* bacteriophage J-1. With the ongoing improvement of AlphaFold2 algorithms and notebooks, there is no doubt that AlphaFold2-driven biological stories will increasingly be reported, which questions the future directions of experimental structural biology.

## Introduction

A major turning point in the field of structural biology was marked when AlphaFold2 - the DeepMind’s machine learning structure prediction algorithm - was made publicly available in mid-2021 (Senior et al., 2020; Jumper et al., 2021). Stunning results produced by AlphaFold2 at the CASP14 (Critical Assessment of Techniques for Protein Structure Prediction) contest indicated that deep learning-based methods are now able to predict protein structures with an accuracy comparable, in most cases, to that of experimental structures (Pereira et al., 2021). This important achievement offers great perspectives to the life science community, including experimented structural biologists as well as untrained biologists, to address challenging biological questions (Goulet and Cambillau, 2021; Tunyasuvunakool et al., 2021). In particular, the AlphaFold Protein Structure Database (https://www.alphafold.ebi.ac.uk), developed by DeepMind and EMBL-EBI, offers open access to structure predictions for the human proteome, for proteins from key model organisms (e.g., *Mus musculus*, *Escherichia coli*, *Arabidopsis thaliana*), and for proteins from organisms related to global health (*Mycobacterium tuberculosis*, *Staphylococcus aureus*, *Trypanosoma cruzi*) (Tunyasuvunakool et al., 2021; Varadi et al., 2022). The number of structures in the AlphaFold database is expected to increase at a dizzying rate, with more than 100 million structures that should be available this year to include most representative sequences from the UniRef90 data set (Varadi et al., 2022).

However, AlphaFold2 is not substituting structural biologists. It is instead becoming a key step of any structural biology project, providing structures that must be properly analyzed. The reliability of predicted protein structures is such that they can be used as starting models for molecular replacement in X-ray crystallography, for the interpretation of cryo-electron microscopy (cryoEM) 3D reconstructions, and for the rational design of mutations and functional assays (Kryshtafovych et al., 2021; Slavin et al., 2021; Alerasool et al., 2022; Ivanov et al., 2022; McCoy et al., 2022; Paul et al., 2022). Moreover, AlphaFold2 can go a step further and predict the structure of large and flexible machineries, thereby circumventing intrinsic limitations of experimental methods (Goulet and Cambillau, 2021).

Here, we present our perspective on the present impact of AlphaFold2 on structural biology. In particular, we illustrate its power in predicting high-confidence structures of samples that cannot be studied by classical experimental approaches, using the *Lactobacillus casei* bacteriophage (phage) J-1 as model (Dieterle et al., 2017). The predicted structure of the whole J-1 host adhesion device provides important information on molecular mechanisms of phage-host interactions, and attests to the present progress of phage structural biology.

## Making the Most out of AlphaFold2 Predictions

**FIGURE 1:**
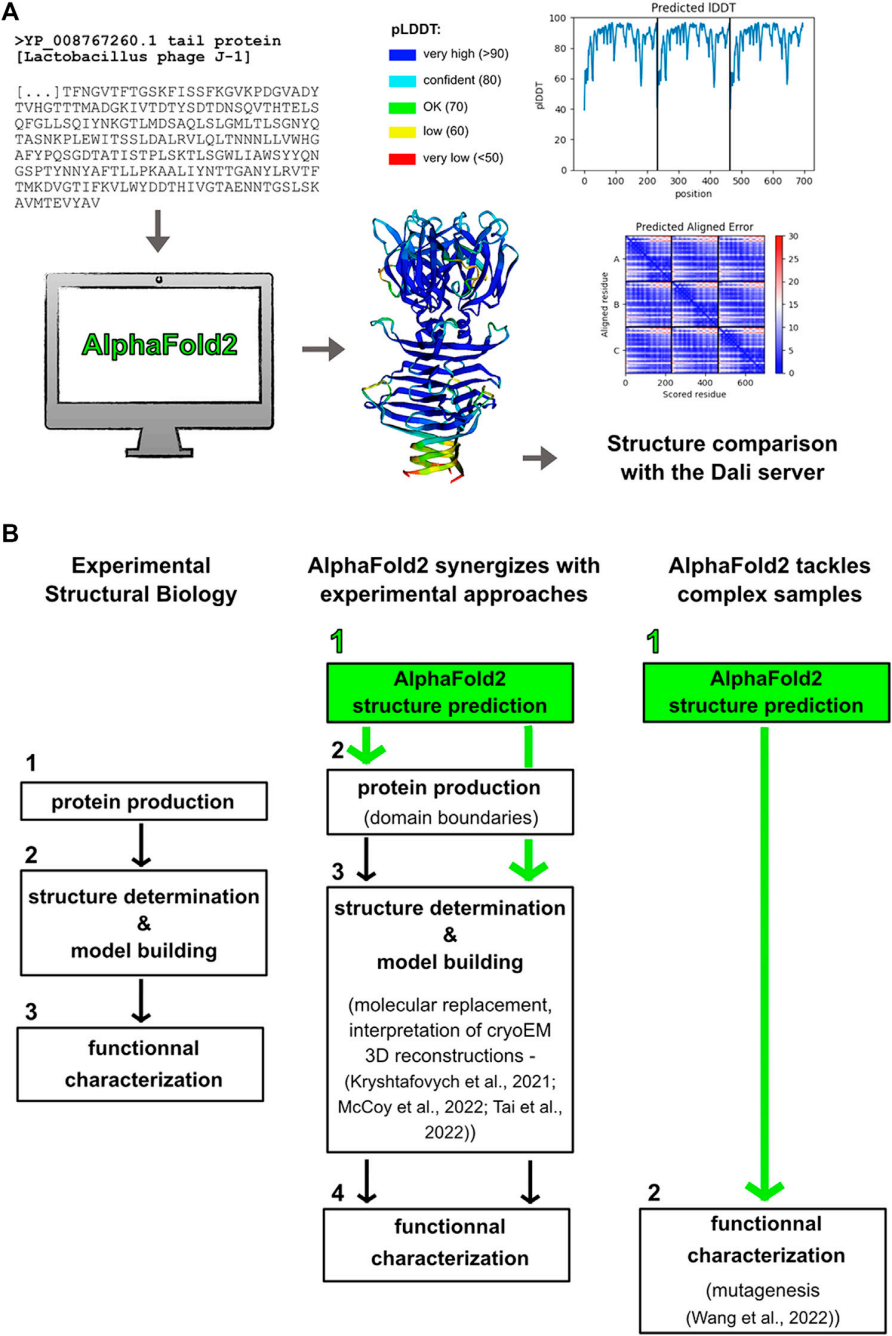
Making the most out of Alphafold2 structure predictions. **(A)** Predicted structures are obtained “simply” by providing their sequences to the program. The pLDDT and PAE scores have to be carefully considered for structure-function analyses. Structure comparison using the Dali server may return structural homologs (Holm, 2020). Here are shown the results obtained for the Tal C-terminal domain (trimeric assembly) from the *L. casei* phage J-1. **(B)** AlphaFold2 is becoming an essential part of any structural biology project. AlphaFold2 can synergize with experimental approaches in that predicted structures can be used to determine domain boundaries for recombinant protein production, to solve crystal structures by molecular replacement, and to interpret cryoEM 3D reconstructions. Moreover, AlphaFold2 can be the only way to get structural models of challenging samples, which can then be used as reliable templates for functional characterization.

AlphaFold2 generates 3D structure predictions for any given sequence (provided the length does not exceed 1,400 amino acids in freely accessible notebooks), which opens new research perspectives for many biologists. However, a solid background in structural biology and protein biochemistry is a critical pre-requisite to properly interpret AlphaFold2 predictions. Basically, AlphaFold2 produces a 3D structure, using an amino acid sequence as input, by taking advantage of evolutionary information inferred from a multiple sequence alignment of homologs. This information provides inter-residue correlations that are translated into contact maps (Jumper et al., 2021). Confidence scores of predicted structures are given by pLDDT (predicted Local Distance Difference Test) per amino acid (Figure 1A). pLDDT values greater than 90% indicate an accuracy in the position of amino acid side chain comparable to that given by experimental crystal structures, while pLDDT values lower than 50% indicate random position. These values are very informative and have to be carefully considered in evaluating the results. The pLDDT values, and hence the prediction reliability, highly depends on the number of aligned sequences. Usually, a minimum of 50–100 aligned sequences is enough to lead to a reasonable prediction. Therefore, drops in pLDDT values in a predicted protein structure correspond to drops in the number of aligned sequences. Moreover, low pLDDT values are often associated with loops and linkers as their sequences are less conserved in length and content as compared to those of the structural core of proteins. Hence, low pLDDT values usually maps well with flexible regions. The low confidence of these regions indicate that the relative orientation of the well-defined adjacent domains has to be interpreted with caution. The Predicted Aligned Error (PAE) is another metric to assess the confidence in the relative position and orientation of different domains (Varadi et al., 2022) (Figure 1A). Also, large ribbon-like structures with low pLDDT values predict intrinsically disordered regions (IDRs) and should not be interpreted as structures (Ruff and Pappu, 2021; Tunyasuvunakool et al., 2021).

## AlphaFold2 is Changing the Way We do Structural Biology

The breakthrough of AlphaFold2, and certainly that of other publicly and freely available deep-learning based prediction methods like RoseTTAFold of the Rosetta suite (Baek et al., 2021), requires us to revisit our way of doing structural biology. This does not mean that experimental approaches are obsolete. Instead, AlphaFold2 expands the structural biology toolbox offering faster and more efficient ways to unravel protein structure-function relationships.

### Alphafold2 Structure Prediction: The Necessary First Step

AlphaFold2 structure prediction should become the first step of any structural biology project (Figure 1B). First of all, this prediction will provide crucial information on domain boundaries to use for successful recombinant protein production, which is the pre-requisite to perform any structural and functional analyses. In the case of a multi-domain protein, drops in the curve plotting pLDDT values over the sequence will indicate sequence stretches to be avoided. Moreover, predicted structures that return structural homologs in the PDB can unveil important functional information, which often cannot be retrieved from protein sequences. This can save months, or even years, of work to determine the first, experimental structure on which all the research project will be based. For instance, catalytic residues of an enzyme can be proposed from an AlphaFold2 structure obtained in few hours (Wang et al., 2022), which then enables to focus experimental efforts on structure determination of the enzyme bound to its substrate for instance, and on its functional characterization (Figure 1B). On the other hand, this prediction can reveal features explaining an unexpected biochemical and functional behavior. For instance, the presence of a loop blockings access to an enzyme active site may be the cause of a loss of activity. In such cases, having this kind of information early in the project will help to save time and efforts that would otherwise be spent in troubleshooting protocol conditions.

### The Interplay Between AlphaFold2 and Experimental Methods

AlphaFold2 provides accurate 3D structure predictions at the atomic level. However, it cannot predict, at the moment at least, ligand binding, folding of IDRs upon partner binding, and multi-partner complex formation. Therefore, structural biology projects can benefit from the synergy between complementary machine-learning predictions and experimental approaches. In particular, AlphaFold2 predictions can be used as starting models for crystallographic phasing by molecular replacement and for model tracing and sequence positioning in cryoEM 3D reconstructions (Figure 1B) (Kryshtafovych et al., 2021; McCoy et al., 2022; Tai et al., 2022). An exciting perspective associated with AlphaFold2 release was the possibility to unlock “dormant’ projects for which the phase problem could not be solved. It will be interesting to assess the impact of AlphaFold2 on the number of PDB entries over the next few years.

## AlphaFold2 Opens up Great Perspectives to Tackle Complex Samples Such as Phages’ Host Adhesion Devices

To date, AlphaFold2 is the only way to get structural and functional insights into complex biological samples. Indeed, some macromolecular assemblies are reluctant to experimental approaches because they cannot produce X-ray-diffracting crystals or particles amenable to 3D reconstructions by cryoEM and single particle analysis (Figure 1B). This is the case of often flexible phages’ host adhesion devices, which are multi-protein assemblies involved in host binding at the onset of viral infection (Veesler and Cambillau, 2011; Goulet et al., 2020). We have chosen to illustrate such AlphaFold2 powerful capabilities with structure prediction of the *L. casei* phage J-1 host-adhesion device.

Tailed phages, and in particular Siphoviridae like J-1, possess the host adhesion device, which is in large part built from conserved components, at the extremity of their flexible tail (Veesler and Cambillau, 2011; Goulet et al., 2020). The core of this machinery always consists of an hexameric Distal Tail protein (Dit), bound on one side to the last hexameric Major Tail Protein (MTP), and on the other side to a trimeric Tail associated lysin (Tal). Dit and Tal may incorporate a variable number of carbohydrate-binding domains (CBM) that trigger phage-host adhesion. On top of these, other modules are often found, such as receptor-binding proteins (RBPs), various fibers, or enzymatic modules. These machineries are often extended and highly flexible in order to explore their surrounding in search of a bacterial host (Linares et al., 2020). From a structural biology view point, due to their flexibility, these machineries can rarely be studied as a whole (for exceptions see (Sciara et al., 2010; Veesler et al., 2012)) but mostly as isolated modules. However, we recently showed that the structure of a long and flexible Tal from the *Oenococcus oeni* phage Vinitor 162 could be predicted with AlphaFold2 (Goulet and Cambillau, 2021).

**FIGURE 2:**
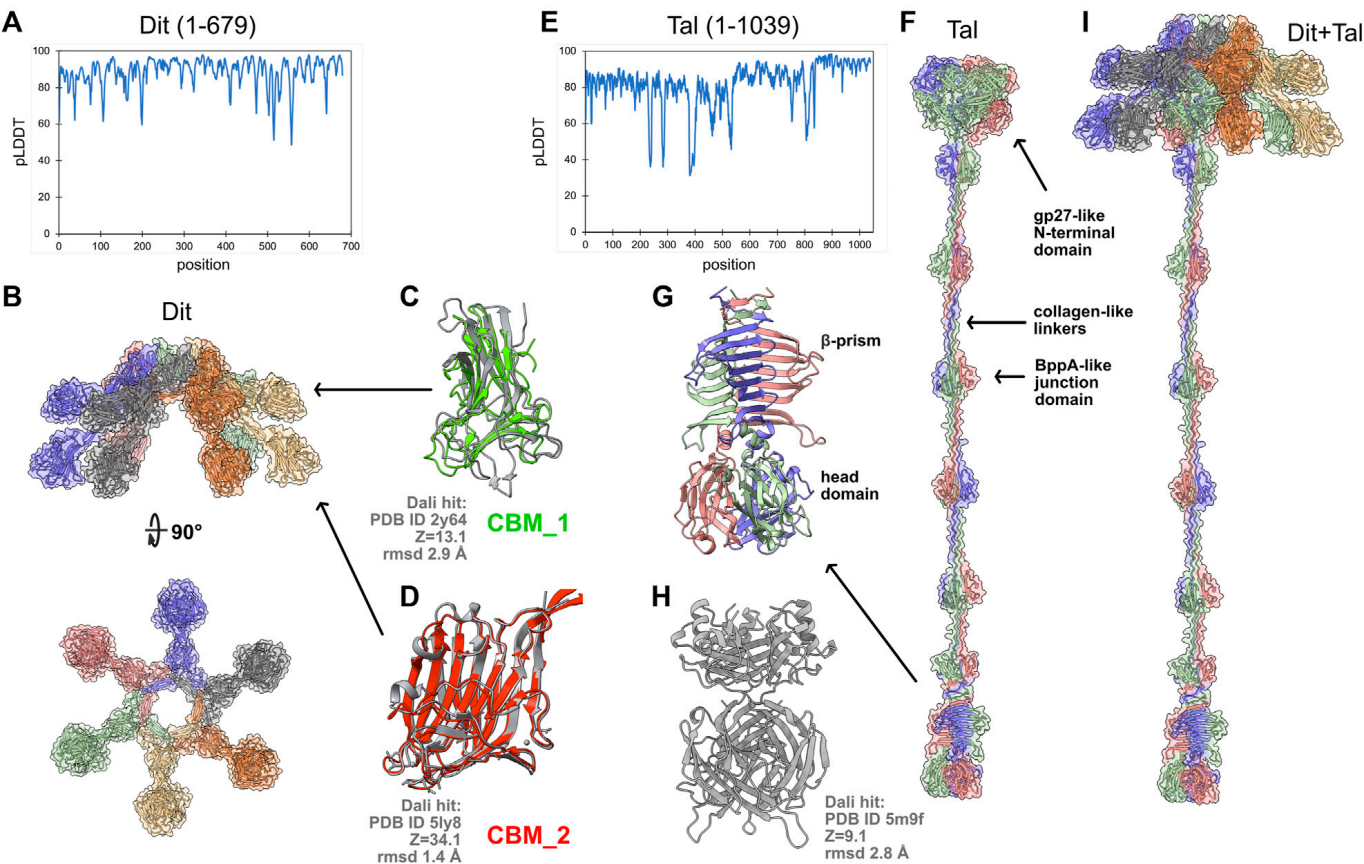
Structure prediction of the host adhesion device from L. casei phage J-1. **(A)** pLDDT plot for the full-length Dit predicted structure. **(B)** Orthogonal views of the Dit hexamer. In the surface and ribbon representations, each monomer is differently colored. **(C)** Superposition of the Dit CBM_1 onto the CBM4-2 from a thermostable *Rhodothermus marinus* xylanase returned as a significant hit using Dali. **(D)** Superposition of the Dit CBM_2 predicted and crystal structures. **(E)** pLDDT plot for the full-length Tal predicted structure. **(F)** Surface and ribbon representations of the Tal trimeric assembly (each monomer is differently colored). **(G)** Close-up view on Tal C-terminal domain, the likely J-1’s RBP. **(H)** The Tal C-terminal domain returned the *Staphylococcus* Virus K RBP as significant hit using the Dali server (Dali statistics apply to the head domain). **(I)** Surface and ribbon representations of the entire J-1 adhesion device. Figures were generated with ChimeraX (Pettersen et al., 2021).

The host adhesion device of J-1 comprises an hexameric ring of Dit (679 amino acids) attached to a trimer of Tal (1,039 amino acids). In 2017, we determined the crystal structure of one of the two CBMs inserted in the Dit, and showed that this domain recognizes and binds to the host cell surface (Dieterle et al., 2017). Also, we fitted this structure, together with the structures of Dit and Tal form the *Lactococcus lactis* phage p2 (Sciara et al., 2010), into a negative staining electron microscopy reconstruction of J-1 host adhesion device to produce a topological model of this multi-component assembly (Dieterle et al., 2017). It is important to note that the long and flexible extension of the Tal could not be resolved in the EM 3D reconstruction. This study is an example of the pre-AlphaFold2 way of doing structural biology to decipher the molecular mechanism of phages’ host-adhesion devices. Interestingly, the AlphaFold2-based approach, which ‘simply’ consists of submitting sequences to appropriate notebooks and comparing predictions to known structures using the Dali server (Figure 1A), provides, more rapidly, an insightful structure of the whole Dit-Tal assembly. We used a Github notebook (https://colab.research.google.com/github/deepmind/alphafold/blob/main/notebooks/AlphaFold.ipynb#scrollTo=XUo6foMQxwS2) to perform the predictions, as it provides a simple and efficient service. To note, this notebook does not use PDB templates (as do ‘true’ AlphaFold2 servers), thereby providing a totally naive structure prediction. The Dit structure was predicted as a full-length monomer and assembled on the *L. lactis* phage p2 Dit hexameric ring with *Coot* (Emsley et al., 2010) (Figures 2A,B; Supplementary Material). Due to memory limitations, a monomer of Tal was first calculated (Figure 2E). Then, Tal segments shorter than 450 amino acids were selected for trimeric structure predictions, and assembled together with *Coot* to reconstitute a complete Tal trimer (Figure 2F). In the Dit predicted structure, two CBMs point out of the central ring by 70 Å (CBM_1) and 40 Å (CBM_2) (Figures 2B–D). Without much surprise, the Dali server reported an excellent match between the CBM_2 predicted and crystal structures (Dieterle et al., 2017) (Z-score of 34.1, root mean square deviation (rmsd) of 1.4 Å; Figure 2D). CBM_1 was found to be similar to the CBM4-2 from a thermostable *Rhodothermus marinus* xylanase (von Schantz et al., 2012) (Figure 2C). The Tal structure, as a trimer, has an overall length of ∼650 Å (Figures 2F,I; Supplementary Material). The N-terminal domain (1-391) pertains to the classical T4 phage gp27 module, found in *Myo-* and Siphoviridae as well as in Type 6 secretion systems (Veesler and Cambillau, 2011). It is followed by six structural domains each mainly composed of three β-strands, and identified by Dali as part of *L. lactis* phage Tuc2009 BppA junction domain (Legrand et al., 2016) (Figure 2F). These domains are linked together by collagen-like extended structures. Following the sixth structural domain, the C-terminus is formed by a short 2-turns β-helix, followed by a 8-stranded β-prism (Figure 2G). Then, after a short helical linker, a compact β-stranded trimeric domain forms the Tal distal end. This last domain resembles the host-binding domain of several phage RBPs (called “heads”), including that of *Staphylococcus* Virus K (Figure 2H), but also those of *L. lactis* phages p2 (Sciara et al., 2010) and TP901-1 (Veesler et al., 2012) or listerial phage PSA (Dunne et al., 2019). These observations make this domain a candidate as the J-1 RBP. Finally, Dit and Tal were assembled with *Coot,* using the phage p2 Dit-Tal assembly as template (Figure 2I), providing an overall view of J-1 host adhesion device. Based on this topology, we propose that J-1 binds to its host using, first, the unique Tal C-terminal RBP, and then, the six functional Dit’s CBM_2 (Dieterle et al., 2017), to secure binding and properly orientate the phage at the host’s surface.

## Discussion

Almost a decade ago, the “resolution revolution” in cryoEM, driven by major technical advances (Kühlbrandt, 2014), dramatically improved the capabilities of structural biology. This field is experiencing another revolution, linked to the impressive improvement of machine-learning structure prediction algorithms like AlphaFold2. Accurate structures can now be predicted for virtually any protein, just from their sequence. AlphaFold2 notebooks are quite convenient and easy to use, thereby allowing structural biologists as well as non-specialists to obtain structures of their favorite samples in few minutes or few hours.

AlphaFold2 is likely to become an essential step of structure determination workflows. Indeed, it synergizes with experimental methods in assisting in the production of suitable samples and the determination/interpretation of experimental structures. Moreover, it represents, to date, the only way to characterize complex machineries at the structural level. In particular, our structure prediction of the host adhesion device from the *L. casei* phage J-1 illustrates the giant step forward that has been made in addressing technically challenging projects. Structural and functional insights can now be obtained on entire, large and flexible machineries. This opens up great perspectives for phage structural biology to better understand molecular mechanisms involved in phage-host interactions. Moreover, AlphaFold2 structure predictions can be used to revisit phage genome annotations and to efficiently characterize the overwhelming number of newly discovered phages, as exemplified by the human gut phageome (Shkoporov et al., 2019).

For the time being, experimental techniques like X-ray crystallography and cryoEM are the only one capable of dealing with conformational dynamics, ligand binding, and complex formation. However, future improvement of machine-learning programs, which might be combined to molecular dynamics calculations, could seriously reduce the prevalence of experimental approaches to decipher protein structure-function relationships.

## Data Availability

The original contributions presented in the study are included in the article/Supplementary Material, further inquiries can be directed to the corresponding authors.
